# Semi-Analytical Large-Strain Bending of Thermoviscoplastic SMP Beams with Plastic Softening

**DOI:** 10.3390/polym18182302

**Published:** 2026-09-20

**Authors:** Hamed Khashabi, Majid Baniassadi, Eunsoo Choi, Mostafa Baghani

**Affiliations:** 1 School of Mechanical Engineering, College of Engineering, University of Tehran, Tehran 14174-66191, Iran; hamed.khashabi@gmail.com (H.K.); m.baniassadi@ut.ac.ir (M.B.); baghani@ut.ac.ir (M.B.); 2 Department of Civil and Environmental Engineering, Hongik University, Seoul 04066, Republic of Korea

**Keywords:** shape memory polymer, semi-analytical formulation, large deformation, thermoviscoplastic constitutive model, pure bending

## Abstract

The accurate prediction of the large-strain thermomechanical response of shape memory polymer (SMP) beams is challenging because geometric nonlinearities must be coupled with temperature-dependent viscoelastic and viscoplastic mechanisms, including plastic softening. This study develops a semi-analytical framework for the large-strain pure bending of thermoviscoplastic SMP beams. The formulation combines finite-deformation bending kinematics with the thermoviscoplastic constitutive model of Zeng et al., while a mapping-based fitting strategy is introduced to approximate the through-thickness distribution of the viscous mechanical stretches. The resulting stress field is obtained by enforcing radial equilibrium in polar coordinates and satisfying the traction-free inner and outer surfaces. The formulation is implemented in MATLAB R2026a and independently verified against a three-dimensional ABAQUS/Explicit model incorporating the same constitutive equations through a user-defined VUMAT. Shape recovery, bending-force recovery, stress distributions, deformation fields, and mesh sensitivity are investigated for two SMP material parameter sets. The semi-analytical and finite element predictions show close agreement throughout the thermomechanical cycles. For example, at the end of the relaxation stage, the predicted inner radii are 27.27 mm and 27.26 mm for the semi-analytical and finite element approaches, respectively, corresponding to a difference of approximately 0.04%. The results demonstrate that the proposed formulation provides an efficient alternative for analyzing large-strain SMP bending while retaining the essential path-dependent thermoviscoplastic behavior.

## 1. Introduction

Shape memory polymers (SMPs) are a class of smart materials capable of maintaining a temporary shape and recovering their original permanent configuration when exposed to an external stimulus. These materials offer several advantages, including large recoverable strains, low density, cost-effectiveness, and biocompatibility [1]. The permanent shape can be programmed during manufacturing or processing [2], while the temporary shape remains stable until activation. SMPs can be triggered by a variety of stimuli, including heat [3,4], electric fields [5,6] and magnetic fields [7]. Moisture and pH variations [8,9] are other interesting choices. Some may also use electromagnetic radiation [10,11], and ultrasound [12,13]. Some SMP systems are also capable of sustaining multiple shape memory cycles with minimal performance degradation [14]. Among these activation mechanisms, thermal stimulation remains the most widely used because of its simplicity and reliability. Owing to their unique combination of properties, SMPs have found applications in biomedical devices [15,16], and textiles [17,18]. SMP metamaterials [19,20] are now a hot topic among researchers. Smart sensors [21,22], and actuators [23,24,25] are also new emerging areas. The growing use of SMPs in engineering applications has motivated the development of constitutive models capable of accurately describing their complex thermomechanical behavior. These models can generally be classified into two categories according to their scale of representation: microscopic and macroscopic approaches [26,27,28,29].

At the microscopic scale, various approaches have been developed to investigate the fundamental mechanisms underlying the shape memory effect. For example, Diani and Gall [30] employed molecular dynamics simulations to study the evolution of polymer chains and molecular interactions responsible for shape memory behavior, while Zhang and Hu [31] used quantum mechanical analyses to investigate the role of hydrogen bonding in SMP copolymers. At the macroscopic scale, SMP constitutive models can generally be classified into three main categories: phase-transition models, thermoviscoelastic models, and hybrid formulations. Phase-transition models represent SMPs as a combination of rubbery and glassy phases whose volume fractions evolve with temperature and loading conditions. As the temperature decreases, the increasing glassy-phase fraction facilitates temporary shape fixation, whereas reheating promotes recovery of the permanent shape. Liu and Gall [32] proposed one of the earliest phase-transition models for small-strain applications, which was later extended to large deformations by Chen and Lagoudas [33] through the incorporation of hyperelasticity.

Thermoviscoelastic models describe the time- and temperature-dependent response of SMPs through viscoelastic constitutive relations with temperature-sensitive material parameters. Tobushi [34] developed an early one-dimensional small-strain formulation based on nonlinear viscoelastic elements, while Diani [35] extended the framework to three-dimensional finite deformations. Subsequently, Westbrook et al. [36] introduced a multi-branch viscoelastic model to capture complex relaxation behaviors over a broad range of time scales. To combine the advantages of different modeling strategies, hybrid formulations have also been proposed. For instance, Guo and Liu [37] developed a constitutive model that integrates phase-transition and viscoelastic concepts, enabling a more comprehensive representation of the thermomechanical response of SMPs. ISV models are particularly effective for describing such path-dependent behavior because they characterize the material response through variables that quantify deviations from thermodynamic equilibrium. Gu [38] developed an ISV-based constitutive model capable of capturing both thermally and mechanically induced shape memory effects. Nguyen et al. [39,40] introduced the concept of fictive temperature to describe non-equilibrium states during the glass transition process. Ostadrahimi et al. [41] proposed a nonlinear thermovisco-hyperelastic framework suitable for multiphysics applications involving large deformations. Xiao et al. [42] developed a multi-branch constitutive model incorporating viscous flow and yielding mechanisms, which was subsequently refined by Zeng et al. [43] through the consolidation of viscoelastic branches and the introduction of an improved thermal expansion formulation. These developments have significantly enhanced the ability of constitutive models to capture the complex thermomechanical and path-dependent behavior of SMPs.

In parallel with constitutive model development, analytical and numerical methods have been proposed to analyze SMP structures subjected to different geometries and loading conditions. Among these structural problems, beam bending has received considerable attention because of its relevance to actuators, biomedical devices, deployable structures, and other engineering applications. Accurate prediction of the bending response is therefore essential for the design and optimization of SMP-based components. Baghani et al. [44] developed an analytical solution based on Euler–Bernoulli beam theory to investigate the bending behavior of SMP beams under small-strain conditions. Their study examined the influence of various loading scenarios and boundary conditions on the thermomechanical response of the structure.

Several analytical and numerical approaches have been developed to investigate the bending behavior of SMP beams. Maleki-Bigdeli [45] and Ghosh and Srinivasa [46] proposed finite element formulations based on von Karman [47] and Euler–Bernoulli beam theories, respectively. Samadi Aghdam et al. [48] and Baghani et al. [49] developed finite element models for multilayer and composite beams, respectively, whereas Eskandari et al. [50] employed Timoshenko beam theory to investigate thick SMP beams. Other researchers examined specialized problems, including vibration behavior in light-activated SMP structures [51], laminated SMP beams [52], and size effects in SMP micro-beams [53]. More recently, Khashabi et al. [54] developed a finite element framework for SMP beams using a viscoplastic constitutive model and verified the results against three-dimensional Abaqus simulations. In addition, Bakhtiyari et al. [55] proposed a semi-analytical solution for the large-strain pure bending of SMP beams based on a thermoviscoelastic constitutive model. This formulation was subsequently extended to multilayer SMP structures through the introduction of additional boundary and continuity conditions [56]. Owing to its computational efficiency and ability to capture large deformations, the semi-analytical framework of Bakhtiyari et al. [55,56] serves as the foundation for the present study.

Despite these developments, an efficient semi-analytical formulation capable of treating finite bending together with thermoviscoplastic effects, plastic softening or other phenomena remains lacking. Existing semi-analytical large-strain formulations for SMP bending have primarily been developed within thermo-viscoelastic or thermovisco-hyperelastic frameworks, whereas thermoviscoplastic beam formulations have generally relied on beam-theory or finite element implementations. In particular, the through-thickness evolution of the viscous deformation gradient introduces an additional difficulty in obtaining the stress field from the radial equilibrium equation. A direct analytical representation of this field is generally unavailable, which limits the applicability of existing large-strain semi-analytical formulations to constitutive models involving viscous flow and plastic softening. Therefore, a computationally efficient formulation that retains finite-deformation kinematics while incorporating thermoviscoplastic behavior and plastic softening is needed. In the present study, a novel semi-analytical solution for the large-strain pure bending of SMP beams governed by a thermoviscoplastic constitutive model (Zeng’s model [43]) with a mapping-based strategy to approximate the through-thickness evolution of viscous stretches is developed, inspired by [55,56]. To verify the proposed formulation, the constitutive model is also implemented in a user-defined VUMAT subroutine within ABAQUS/Explicit. The semi-analytical predictions are compared with finite element simulations for shape recovery, force recovery, and stress distributions, demonstrating the accuracy and robustness of the proposed approach.

## 2. A Brief Review of the Constitutive Model

**Figure 1:**
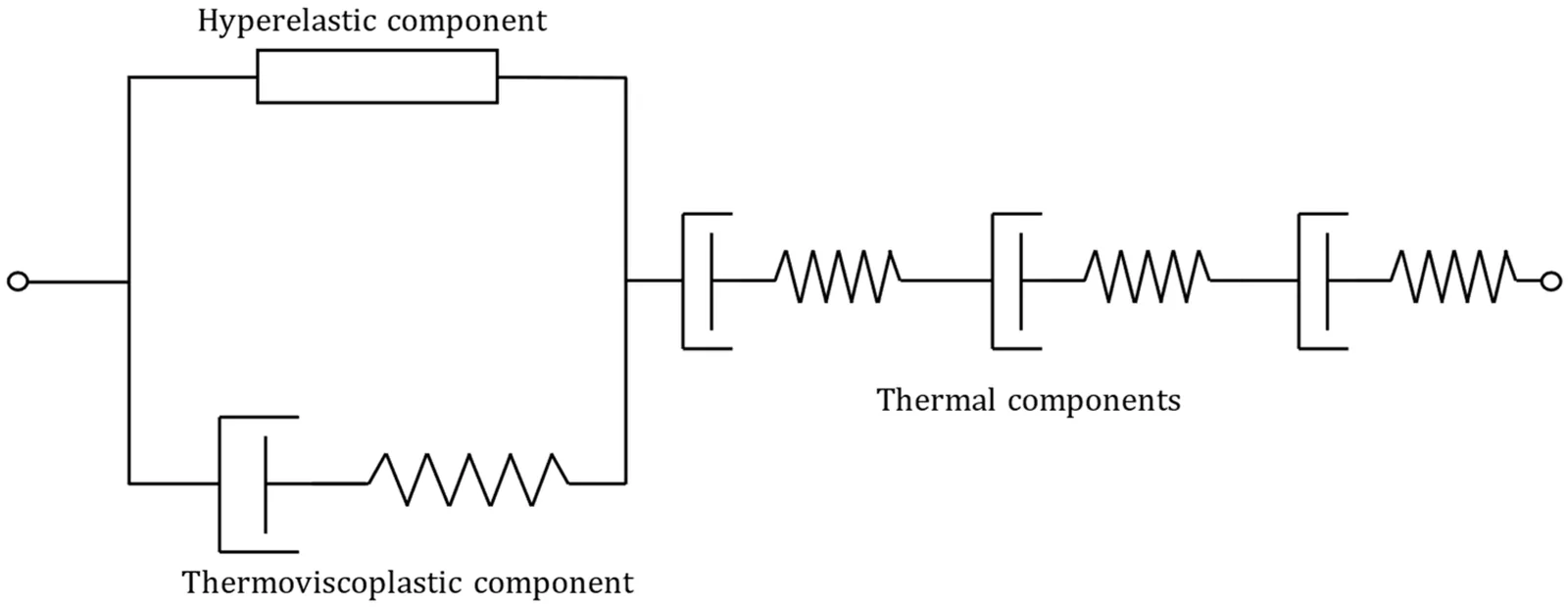
Thermoviscoplastic constitutive model.

This section briefly reviews the thermoviscoplastic constitutive model adopted in this study. The model employs internal state variables to capture the thermomechanical response of shape memory polymers and path-dependent phenomena such as plastic softening. It is based on the framework proposed by Zeng et al. [52], with modifications to the stress-relaxation behavior to improve the prediction of shape and stress recovery. The corresponding rheological representation for amorphous shape memory polymers is shown in Figure 1.

The deformation gradient F is decomposed into thermal and mechanical components, which are further split into equilibrium, hyperelastic parts and non-equilibrium, viscoelastic parts. (The bold format is intended to denote tensors.)

(1) $$F=F_{T}F_{M}=F_{T}F_{M}^{e}F_{M}^{v}$$

The Cauchy stress is divided into three distinct parts:

(2) $$\sigma =\sigma_{eq}+\sigma_{neq}+P$$

The Cauchy stress is composed of an equilibrium component $\left(\sigma_{eq}\right)$, a non-equilibrium component $\left(\sigma_{neq}\right)$, and a volumetric component $\left(P\right)$. The equilibrium part of the Cauchy stress is defined as

(3) $$\sigma_{eq}=\frac{\mu^{eq}}{3J}\frac{T}{T_{g}}\frac{\lambda_{L}}{\lambda_{eff}}L^{-1}\left(\frac{\lambda_{eff}}{\lambda_{L}}\right)(\bar{B_{M}}-\frac{1}{3}{\bar{I}}_{M1}I)$$

Here, ${\bar{B}}_{M}=J_{M}^{-\frac{2}{3}}\;B_{M}$, $J_{M}=det\left(F_{M}\right)$, and ${\bar{I}}_{M1}=\;{\bar{B}}_{M}:\;I$. The function $L\left(u\right)=coth\left(u\right)-\;u^{-1}$ is the Langevin function, $\lambda_{eff}$ is the effective network chain stretch, $\lambda_{L}$ is the locking stretch, and $\mu^{eq}$ describes the specific stiffness of the equilibrium component. The non-equilibrium stress response can be described by a modified Neo-Hookean model as follows:

(4) $$\sigma_{neq}=\frac{\mu^{neq}}{J}(\bar{B_{M}^{e}}-\frac{1}{3}{\bar{I}}_{M1}^{e}I)$$

In this formulation, ${\bar{B}}_{M}^{e}={\left(J_{M}^{e}\right)}^{-\frac{2}{3}}B_{M}^{e}$, $J_{M}^{e}=det\left(F_{M}^{e}\right)$, ${\bar{I}}_{M1}^{e}=\;{\bar{B}}_{M}^{e}:\;I$, and $\mu^{neq}$ is the specific stiffness for the relaxation mechanisms, which is equivalent to $G=\frac{E_{s}}{2+2\mu }$, the Lamé constant, considering $E_{s}$ and μ as storage modulus and the Poisson ratio, respectively.

Finally, the volumetric stress can be defined as

(5) $$P=\frac{\kappa }{J}(J_{M}^{2}-1)I$$

where *κ* is the bulk modulus in the undeformed configuration. It should be noted that for the semi-analytical formulation, a Lagrange multiplier is used for volumetric stress due to the incompressibility assumption. For further information about the model’s evolution law and material parameter values, see Appendix A.

## 3. Development of Semi-Analytical and Numerical Solution

**Figure 2:**
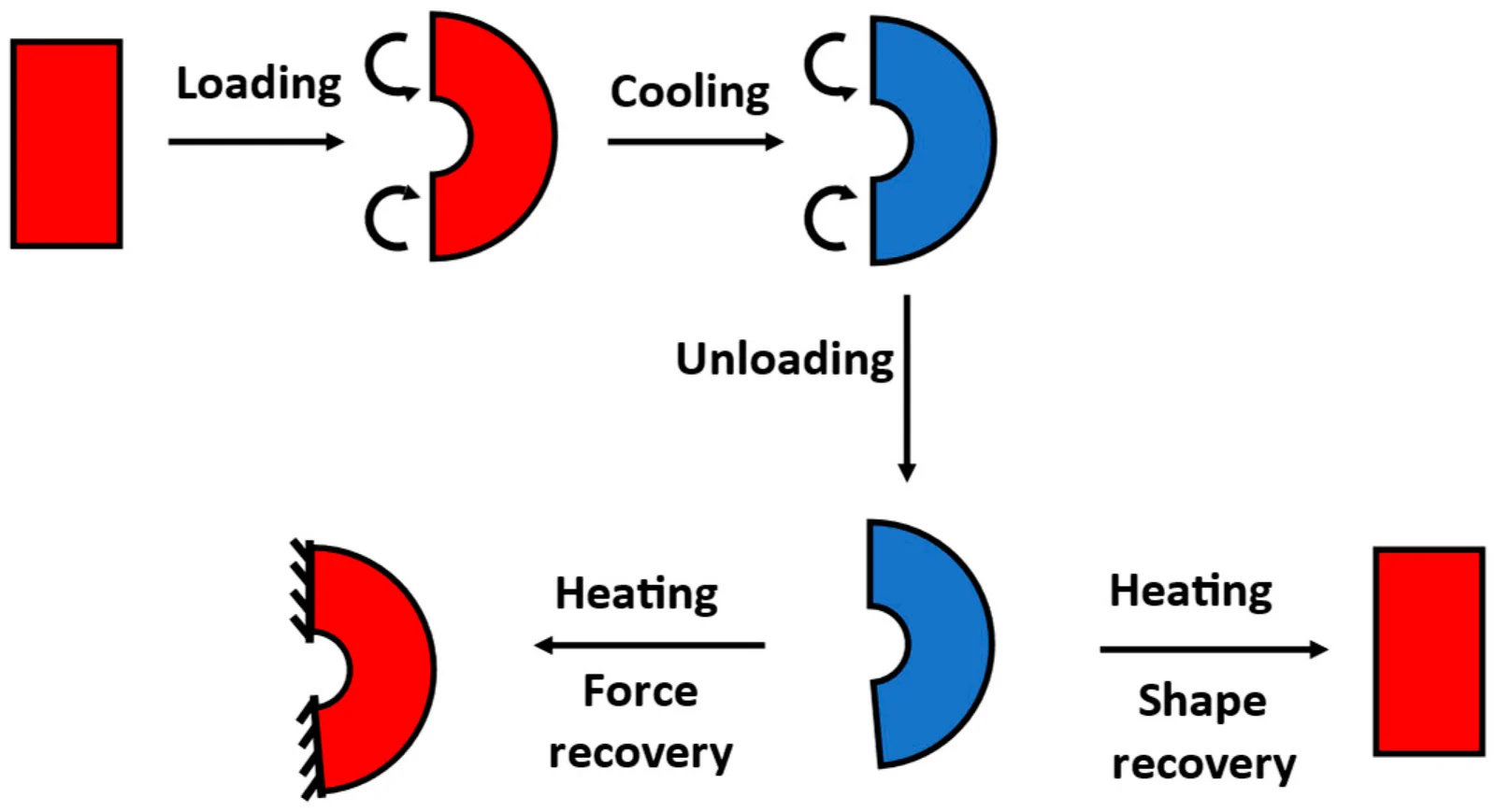
Shape and force recovery cycle.

This section presents the semi-analytical formulation and numerical implementation developed for the analysis of SMP beams under pure bending using the thermoviscoplastic constitutive model. The solution procedure, discretization strategy, and numerical algorithms employed in the analysis are described. The shape and force recovery cycle solved in this work is illustrated in Figure 2.

### 3.1. Kinematics

**Figure 3:**
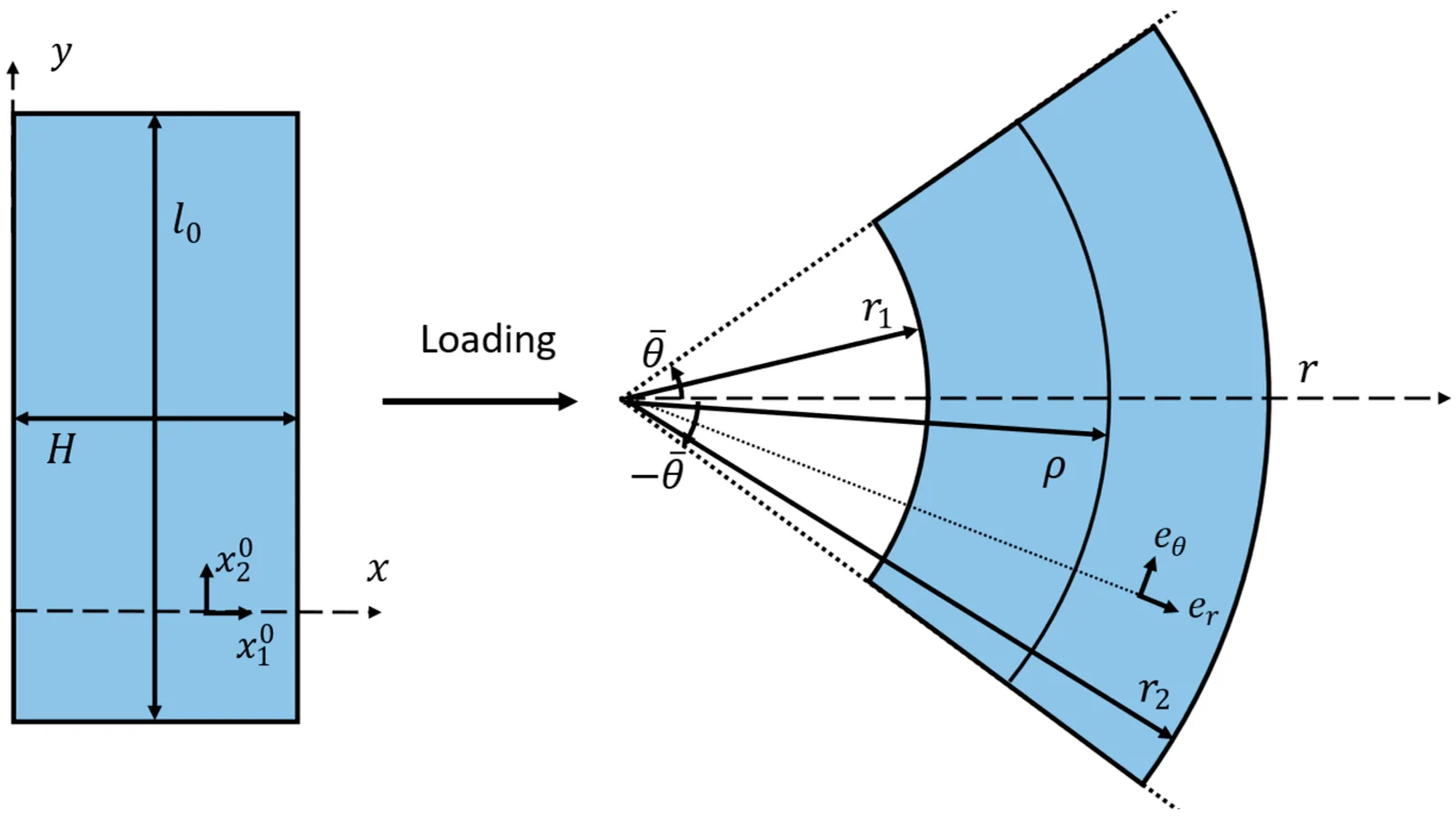
Deformation of the beam under pure bending.

This section presents the kinematic relations governing the large-deformation pure bending of the beam. The formulation assumes that an initially rectangular beam subjected to pure bending under plane-strain conditions deforms into a segment of a hollow circular arc. Under the plane-strain assumption, the stretch in the out-of-plane direction remains unity throughout the deformation. In addition, the material is assumed to be incompressible. For isothermal deformation, incompressibility implies conservation of the beam cross-sectional area, which forms the basis of the kinematic formulation. As illustrated in Figure 3, the initial rectangular beam deforms into a curved configuration with cylindrical geometry. Consequently, the deformed configuration is conveniently described using a polar coordinate system.

The material position in the initial configuration is defined in the Cartesian coordinate system as $\left(x_{1},x_{2},x_{3}\right)$ with unit vectors $\left(e_{1},e_{2},e_{3}\right)$. After bending, the coordinates are described in the polar system as (r,θ,z) with corresponding unit vectors $\left(e_{r},e_{\theta },e_{z}\right)$. The domain of points in the initial configuration is as follows:

(6) $$-\frac{h_{0}^{\left(n\right)}}{2}\leq x_{1}\leq \frac{h_{0}^{\left(n\right)}}{2},\;-\frac{l_{0}}{2}\leq x_{2}\leq \frac{l_{0}}{2},\;-\infty \leq x_{3}\leq \infty$$

After deformation, the domain of points in the new coordinate system becomes

(7) $$r_{1}\leq r\leq r_{2},\;-\bar{\theta }\leq \theta \leq \bar{\theta },\;-\infty \leq z\leq \infty$$

The new position can be expressed as a function of the initial position, as follows:

(8) $$r=r\left(x_{1}\right),\;\theta =\theta \left(x_{2}\right)=\frac{x_{2}}{\rho }\;(\bar{\theta }=\frac{J^{\frac{1}{2}}l_{0}}{2\rho }),\;z=x_{3}$$

where $l_{0}$ is the beam’s original length, $\overset{ˉ}{\theta }$ is the bending angle, ρ is the curvature radius on which the length of the curvature is equal to $l_{0}$, and $r_{1}$ and $r_{2}$ are the inner and outer radii in the bent state, respectively. The deformation gradient is therefore constructed as follows:

(9) $$F=\frac{dr}{dx_{1}}e_{r}⨂e_{1}+r\frac{d\theta }{dx_{2}}e_{\theta }⨂e_{2}+e_{z}⨂e_{3}$$

where ⊗ denotes the dyadic product. Based on the above equation, the principal stretches in this problem are defined as follows:

(10) $$\lambda_{r}=\frac{dr}{dx_{1}}\;,\;\lambda_{\theta }=\frac{r}{\rho }\;,\;\lambda_{z}=1$$

As mentioned, the thermal deformation gradient is expressed as FT=JT13I. Applying the multiplicative decomposition for F, the mechanical deformation gradient is obtained:

(11) $$F_{M}=F_{T}^{-1}F$$

The material is considered incompressible; hence, $F_{M}=1$ ($J=J_{T}$). Consequently, $r\left(x_{1}\right)$ can be obtained as

(12) $$\frac{dr}{dx_{1}}=\frac{J\rho }{r}\;\to r\left(x_{1}\right)=\sqrt{2J\rho x_{1}+C}$$

where C is the integration constant.

### 3.2. The Stress Relations

The stress for the thermoviscoplastic constitutive model considering incompressibility is defined as

(13) $$\sigma =\sigma_{eq}+\sigma_{neq}+PI$$

(14) $$\sigma_{eq}=\frac{\mu^{eq}}{3J}\frac{T}{T_{g}}\frac{\lambda_{L}}{\lambda_{eff}}L^{-1}\left(\frac{\lambda_{eff}}{\lambda_{L}}\right)(\bar{B_{M}})$$

(15) $$\sigma_{neq}=\frac{\mu^{neq}}{J}(\bar{B_{M}^{e}})$$

(16) $$P=P_{eq}+P_{neq}+p$$

where P is the volumetric stress, which consistss of equilibrium, non-equilibrium parts which is obtained via equilibrium equations, and a constant part which is determined with boundary conditions. The terms equilibrium and non-equilibrium parts of volumetric stress are derived from equilibrium and non-equilibrium parts of the total stress, respectively. In order to obtain P, the radial equilibrium equation [57] in cylindrical coordinates is utilized as follows:

(17) $$\frac{\partial \sigma_{rr}}{\partial r}+\frac{\sigma_{rr}-\sigma_{\theta \theta }}{r}+\frac{1}{r}\frac{\partial \sigma_{r\theta }}{\partial \theta }+\frac{\partial \sigma_{rz}}{\partial z}=0\;(\sigma_{r\theta }=0,\;\sigma_{rz}=0)$$

Stress components in radial and tangential directions are substituted into the above equation and the obtained differential equation is solved to find the equilibrium and non-equilibrium parts of volumetric stress. The equilibrium and non-equilibrium volumetric stresses are defined as follows:

(18) $$P_{eq}=\frac{\mu^{eq}}{3J}\frac{T}{T_{g}}\frac{\lambda_{L}}{\lambda_{eff}}L^{-1}\left(\frac{\lambda_{eff}}{\lambda_{L}}\right)\left(\int \frac{1}{r}\left({\left(\frac{r}{\rho }\right)}^{2}-{\left(\frac{\rho J}{r}\right)}^{2}\right)dr-{\left(\frac{\rho J}{r}\right)}^{2}\right)$$

(19) $$P_{eq}=\frac{\mu^{eq}}{3J}\frac{T}{T_{g}}\frac{\lambda_{L}}{\lambda_{eff}}L^{-1}\left(\frac{\lambda_{eff}}{\lambda_{L}}\right)\left({\frac{1}{2}\left(\frac{r}{\rho }\right)}^{2}-\frac{1}{2}{\left(\frac{\rho J}{r}\right)}^{2}\right)$$

(20) $$P_{neq}=\frac{\mu^{neq}}{J}\left(\int \frac{1}{r}\left({\left(\frac{r}{\rho {f_{v}}_{\theta }}\right)}^{2}-{\left(\frac{\rho J}{r{f_{v}}_{r}}\right)}^{2}\right)dr-{\left(\frac{\rho J}{r{f_{v}}_{r}}\right)}^{2}\right)$$

where ${f_{v}}_{\theta }$ and ${f_{v}}_{r}$ are defined as the stretch magnitude functions for the viscous component of the deformation gradient along the $e_{\theta }$ and $e_{r}$ directions, respectively. Structurally, each of these functions is similar to the stretch defined in the preceding subsection, with the addition of a mapping procedure. This mapping enables the function, originally defined at the integration points of the previous computational step, to be evaluated at the new points of the current step.

With the established mapping, the functions ${f_{v}}_{\theta }$ and ${f_{v}}_{r}$ can be formulated as follows:

(21) $${f_{v}}_{r}=\frac{c_{r}\rho }{\bar{\theta }\sqrt{r^{2}-{\left(r_{1}\right)}^{2}+{\left({r_{1}^{\prime }}_{r}\right)}^{2}}}$$

(22) $${f_{v}}_{\theta }=\frac{c_{\theta }\sqrt{r^{2}-{\left(r_{1}\right)}^{2}+{\left({r_{1}^{\prime }}_{\theta }\right)}^{2}}}{\rho }$$

For further reading on mapping and obtaining the non-equilibrium part of the volumetric stress, see Appendix B.

### 3.3. Formulating the Boundary Conditions

**Figure 4:**
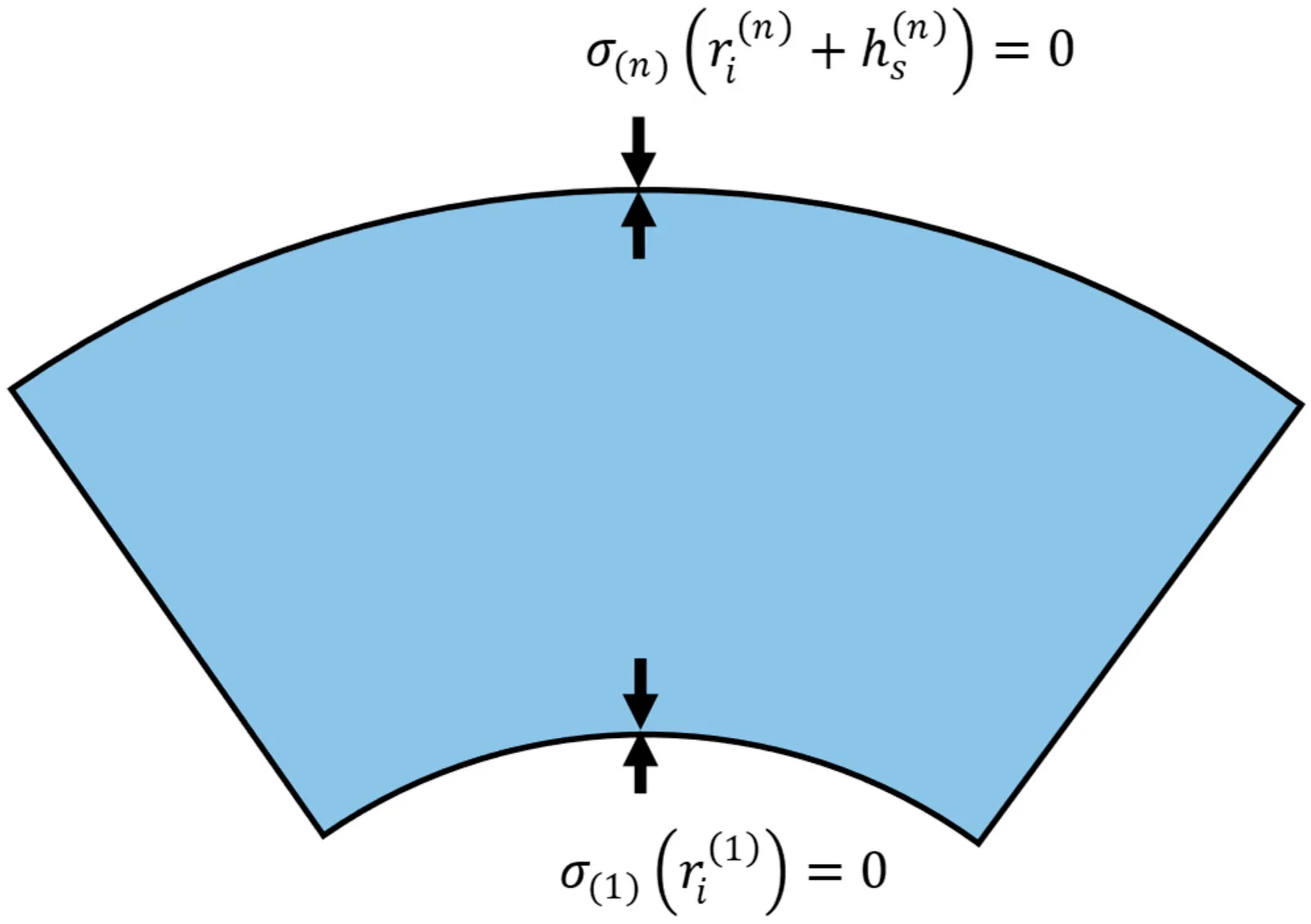
Boundary conditions of the problem.

In this subsection, the boundary condition equations required to determine the problem’s unknowns are discussed. For a known bending angle problem, the unknowns of the problem are the constant volumetric stress and the constant C (ρ can be calculated using $\rho =\frac{J^{\frac{1}{3}}l_{0}}{2\bar{\theta }}$). Consequently, there are two unknowns, necessitating two independent equations for a solution. As illustrated in Figure 4, the stress in the radial direction at the two inner and outer radius boundaries must be zero. By formulating the equations corresponding to these boundary conditions, a set of two equations is obtained, which is sufficient to solve the problem.

For an unknown bending angle problem, besides C and constant volumetric stress, the bending angle $\bar{\theta }$ should also be determined. For this kind of problem, besides the two boundary conditions, another equation (the bending moment force equals zero) should be added to the existing system of equations (known bending angle) to determine the three unknowns using the three obtained equations.

(23) $$\sigma_{rr}(r_{1})=0\;,\;\sigma_{rr}(r_{2})=0$$

The bending moment for this structure is determined as

(24) $$M=\int \sigma_{\theta \theta }rdr$$

### 3.4. Solving Procedure

In this section, the solution procedure for two distinct loading scenarios: fixed-end angular loading (known $\bar{\theta }$) and free-end loading (unknown $\bar{\theta }$) are discussed. The primary difference between these two cases lies in the number of unknowns in the problem. As mentioned in the previous section, the fixed-end angular condition involves two unknowns. However, in the free-end condition, the bending angle, $\overset{ˉ}{\theta }$, is not predetermined. This parameter is therefore added to the set of unknowns and must be determined during the solution process. This addition increases the number of equations (bending moment equals zero) and unknowns in the free-end case, resulting in more computational complexity. The accuracy and validity of the proposed solution methods for both loading types have been verified by comparing the results with those obtained from the explicit FEM. This comparison demonstrates that the presented semi-analytical solution possesses sufficient accuracy and can be reliably used to analyze the beam’s behavior under various loading conditions. For an illustration of the flowchart of the solving procedure, see Appendix C.

### 3.5. The VUMAT Implementation

In this section, the development and implementation of the VUMAT user subroutine to define the constitutive model within the ABAQUS software is discussed. The problem is solved using the VUMAT subroutine in an explicit framework. At each time increment, various parameters are computed, including strain, stress, material temperature, nodal forces, nodal velocities, and accelerations, as well as internal state variables. This computational process proceeds sequentially and iteratively until the final time increment is reached. At every increment, F is obtained by calling it from ABAQUS Explicit, and then $F_{M}$, $F_{v}$, $F_{e}$, and $F_{T}$ are obtained by including their previous amounts and calculating stress; then, internal variables and viscous deformation are updated based on their old values. For further reading about the VUMAT subroutine, see Appendix D.

## 4. Results and Discussion

In this section, the results obtained from the proposed method and the explicit FEM are illustrated and compared. By comparing these two approaches, the validity of both methods can be verified. Also, mesh-dependency of the explicit finite element solution is evaluated. This section includes results related to bending force recovery, shape recovery, stress distributions along the radial axis, stress relaxation at different temperatures, and mesh-dependency of the explicit FEM.

The cycle used to investigate shape recovery was designed as follows: In the first step, the beam is subjected to a bending load and deforms to an angle of 180 degrees over 100 s. It is then held in this configuration for another 100 s to allow the material to relax. Subsequently, the structure’s temperature is decreased from the initial 343 K to 273 K over 500 s to fix the temporary shape. After cooling, the structure is unloaded and both ends of the beam are not constrained for 100 s. Finally, shape recovery to the original configuration occurs as the temperature is increased from 273 K to 373 K over 1000 s.

**Figure 5:**
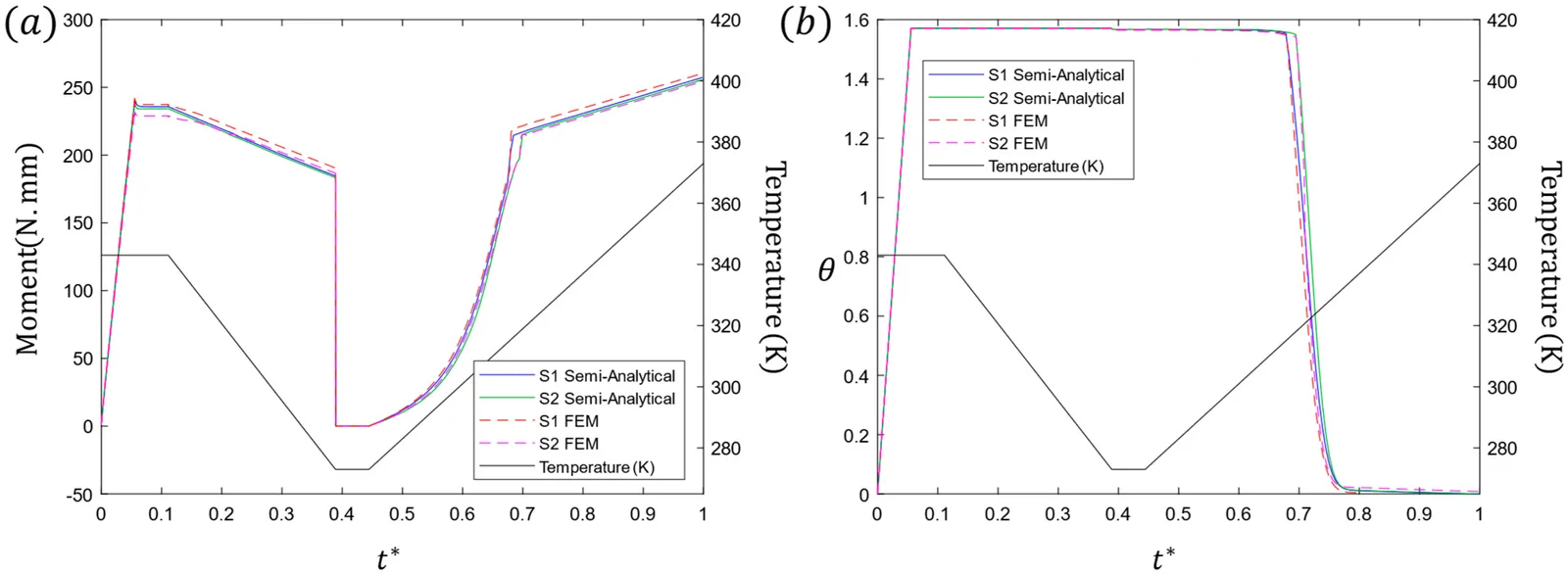
Force recovery (**a**) and Shape recovery (**b**) ($t^{*}=\frac{time}{1800s}$).

To examine the bending force recovery phenomenon, the same cycle as for shape recovery was followed with one key difference. After the unloading stage, where the beam ends are released, the ends are constrained again. The recovery of the bending moment—required to deform the structure from its initial state to the high-temperature temporary shape—is then measured as the structure’s temperature increases. Figure 5 presents the results for shape recovery and bending force recovery in shape memory polymers 1 and 2. The agreement observed in Figure 5 demonstrates that the proposed mapping-based treatment of the through-thickness viscous stretches is capable of transferring the local constitutive evolution into the global bending response.

**Figure 6:**
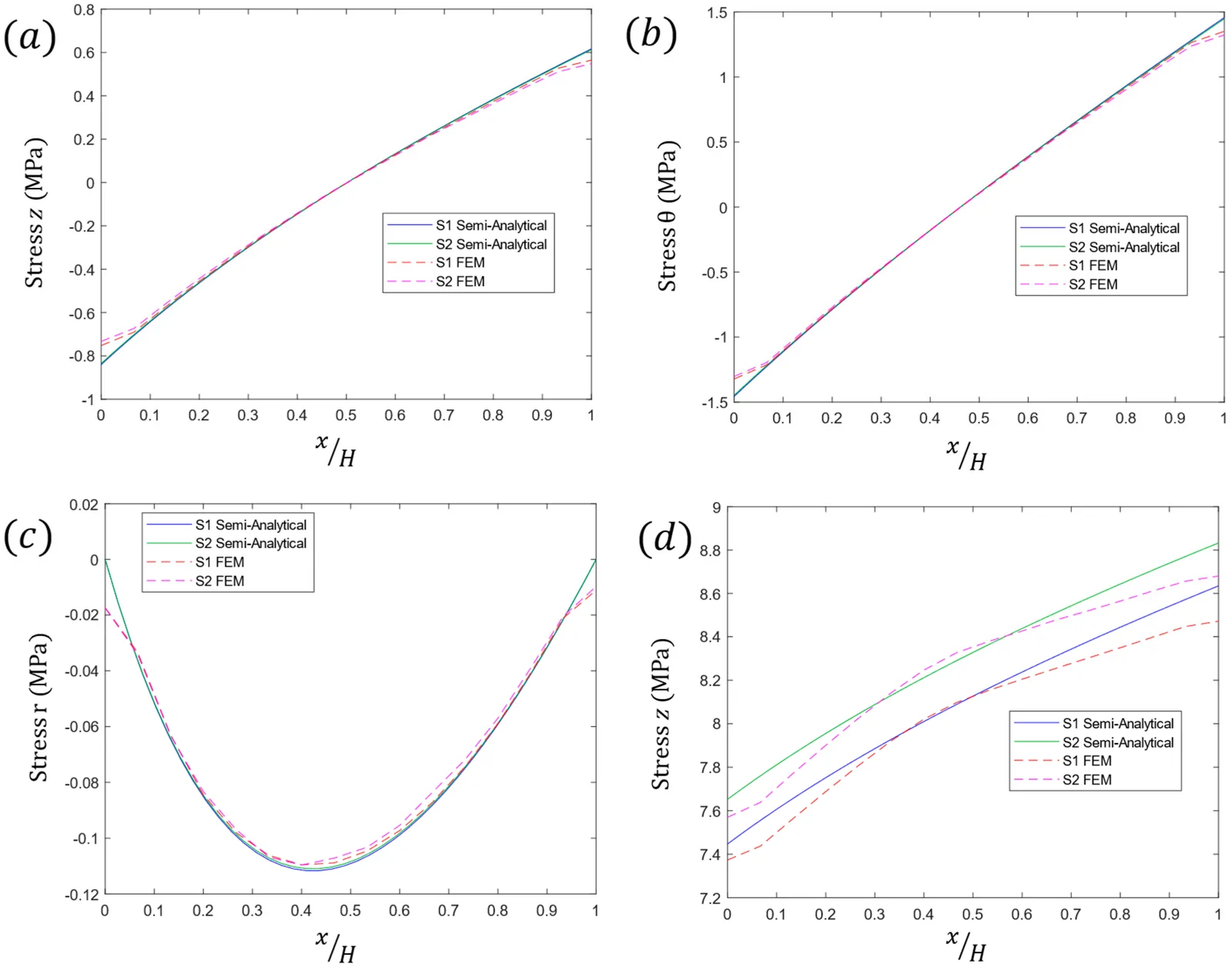
Principal stresses along the radial axis (initial material position through the beam height) for the single-layer beam: at the end of the post-loading relaxation phase (**a**–**c**); at the end of the cooling phase (**d**). (**a**) z-direction stress, (**b**) θ-direction stress, (**c**) r-direction stress, (**d**) z-direction stress (H=10 mm).

**Figure 7:**
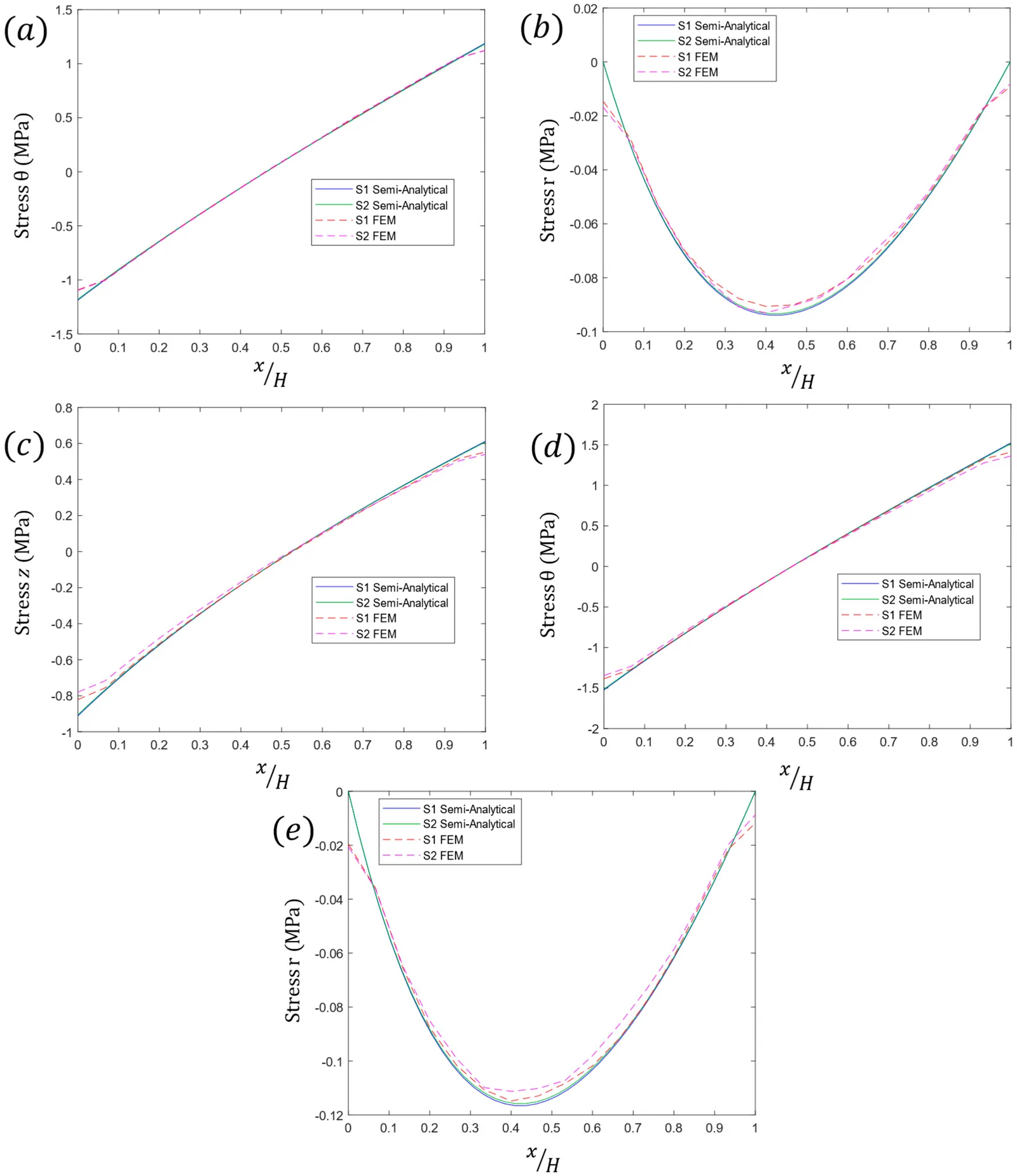
Principal stresses along the radial axis (initial material position through the beam height) for the single-layer beam: at the end of the cooling phase (**a**,**b**); and at the end of the bending force recovery cycle (**c**–**e**). (**a**) θ-direction stress; (**b**) r-direction stress; (**c**) z-direction stress; (**d**) θ-direction stress; (**e**) r-direction stress (H=10 mm).

Figure 6 and Figure 7 illustrate the distribution of principal stresses relative to the initial through-thickness material position for SMPs 1 and 2. These profiles were recorded at three critical moments: the end of the post-loading relaxation step, the end of the cooling step, and the end of the bending force recovery cycle.

**Figure 8:**
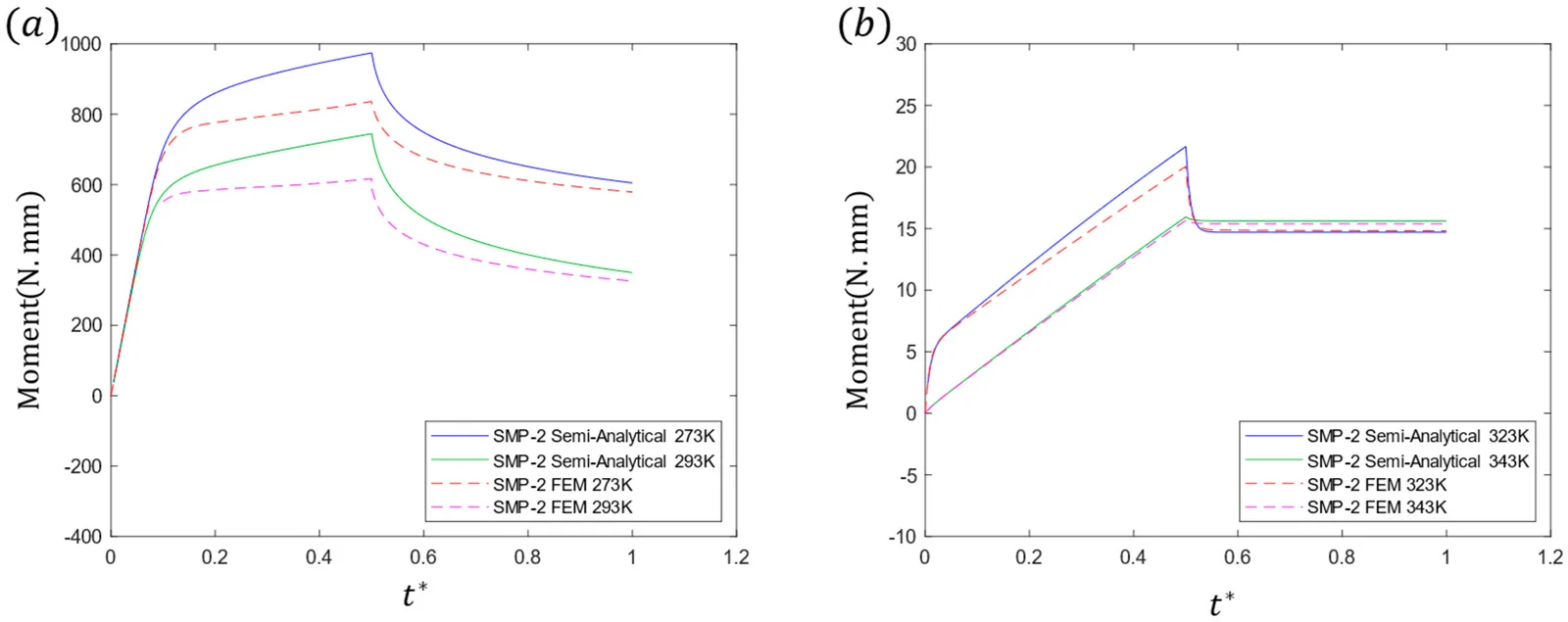
Bending moment results of relaxation loading at different temperatures for (**a**) glassy state (temperature below $T_{g}$) and (**b**) rubbery state (temperature above $T_{g}$) ($t^{*}=\frac{time}{600s}$).

As fundamental solid mechanics principles would dictate, the radial stress at the top and bottom surfaces of the beam must be zero. The results from the semi-analytical method satisfy this boundary condition accurately. In contrast, the explicit FEM exhibits minor deviations from this theoretical value due to numerical limitations and computational approximations. Overall, the comparison of the results from the two methods demonstrates the accuracy and reliability of the proposed semi-analytical approach and the explicit FEM. Figure 8 presents the stress relaxation simulation results for shape memory polymer 2 at various temperatures. In this simulation series, the loading phase was conducted over 300 s, followed by an additional 300 s where the specimen underwent relaxation conditions. These results clearly demonstrate that the proposed model effectively simulates the behavior of this structure across a broad temperature range.

A closer examination of these discrepancies reveals that the primary cause of the mismatches is a deviation in the evolution of the material’s viscous component from the predictions made by the defined fitting functions. In other words, at temperatures much lower than $T_{g}$, the actual structural behavior diverges somewhat from the defined model, resulting in the observed differences in the results. The main reason for this discrepancy is the difference in flow nature at temperatures above and below $T_{g}$. At temperatures above $T_{g}$, the flow is mainly viscous flow, while at temperatures below $T_{g}$, the viscous flow is negligible but the plastic softening effect plays the main role in flow due to high stress.

**Figure 9:**
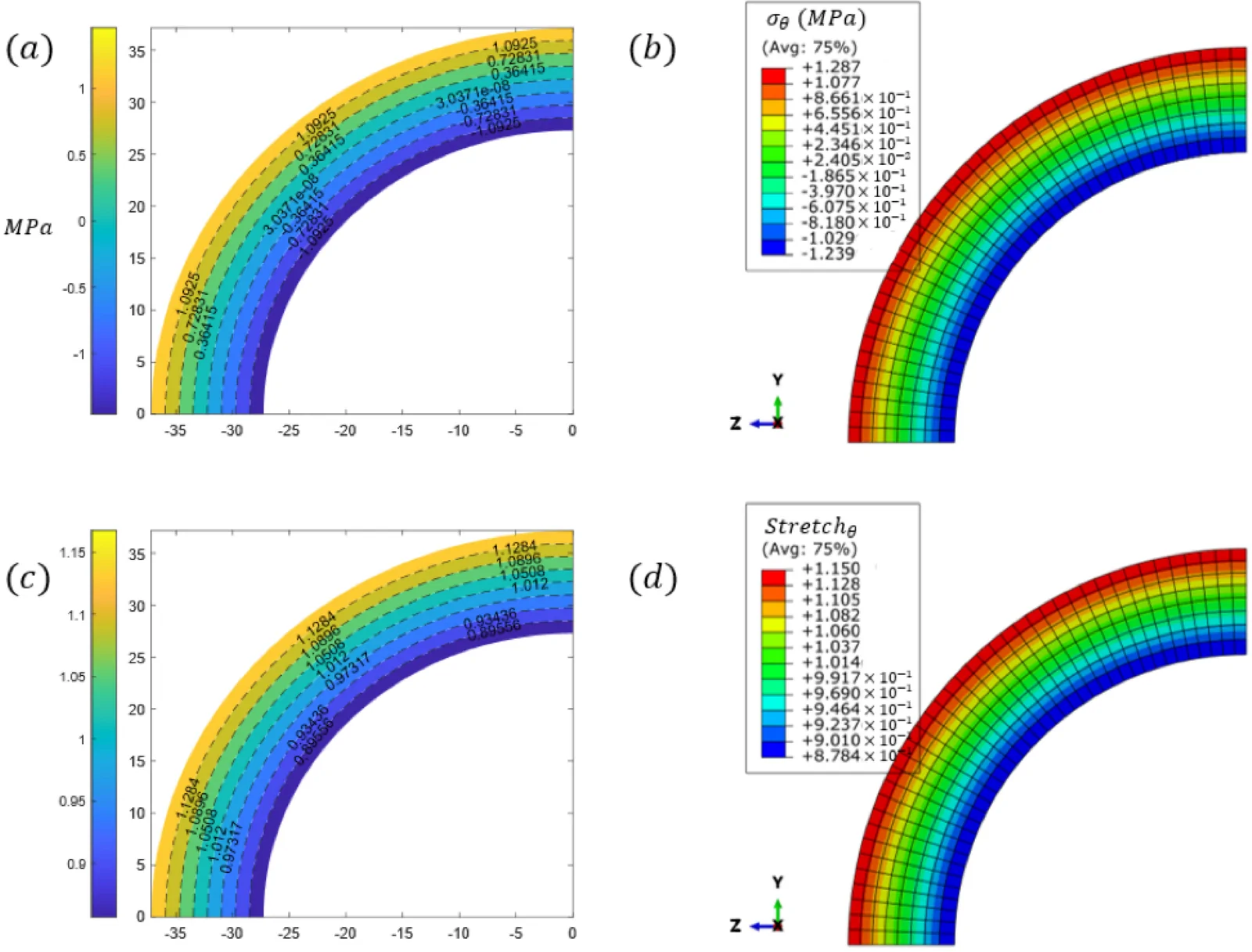
The stress (**a**,**b**) and stretch (**c**,**d**) contours of the SMP−1 at the end of the relaxing step, as obtained using the semi-analytical (**a**,**c**) and numerical (**b**,**d**) approaches.

Figure 9 presents the stress and stretch contours in the deformed configurations of the SMP-1 at the end of the relaxing step, as derived through semi-analytical and numerical approaches. The inner radius obtained from the semi-analytical formulation is 27.27 mm, compared with 27.26 mm from the finite element solution. The corresponding relative difference is only 0.037%, indicating excellent agreement in the predicted deformed geometry.

For further reading about the results of principal stretches and mesh-dependency of the explicit analysis, see Appendix E.

## 5. Conclusions

A semi-analytical formulation was developed for the large-strain pure bending of thermoviscoplastic shape memory polymer beams with plastic softening. The main contribution of the proposed framework is the integration of a thermoviscoplastic constitutive description with finite-deformation bending kinematics while retaining the computational efficiency of a semi-analytical solution.

The formulation introduces a mapping-based approximation for the through-thickness viscous stretches, which enables the radial equilibrium equations to be evaluated and the corresponding volumetric stress field to be determined. The approach therefore provides a practical means of incorporating spatially varying viscous deformation into a large-strain analytical framework without requiring a full finite element discretization.

Independent ABAQUS/Explicit simulations using a user-defined VUMAT were employed to verify the formulation. The semi-analytical and finite element solutions showed close agreement for shape recovery, force recovery, stress distributions, and deformation fields. As one quantitative example, the predicted inner radius at the end of the relaxation stage differed by only approximately 0.037% between the two approaches. The convergence and mesh-sensitivity studies further demonstrated the numerical robustness of the proposed procedure.

The temperature-dependent relaxation results also revealed an important limitation of the present formulation. The discrepancy between the semi-analytical and finite element predictions increases in the glassy-state/transition-temperature regime, where the viscous deformation evolves more rapidly and the adopted spatial fitting functions become less accurate. This observation provides a clear direction for improving the formulation through adaptive or higher-order representations of the through-thickness viscous stretches.

The proposed framework provides an efficient computational tool for investigating large-strain SMP bending and can be extended to multilayer structures, more complex loading paths, and additional internal-variable mechanisms. Its low computational cost also makes it potentially useful for inverse analysis and parameter identification. Nevertheless, experimental validation of the complete large-strain bending problem remains necessary. Future work will therefore focus on controlled thermomechanical bending experiments to independently assess the predictive capability of the formulation and to determine whether improved fitting functions or additional constitutive parameters are required near the glass-transition region.

## Figures and Tables

**Figure A1:**
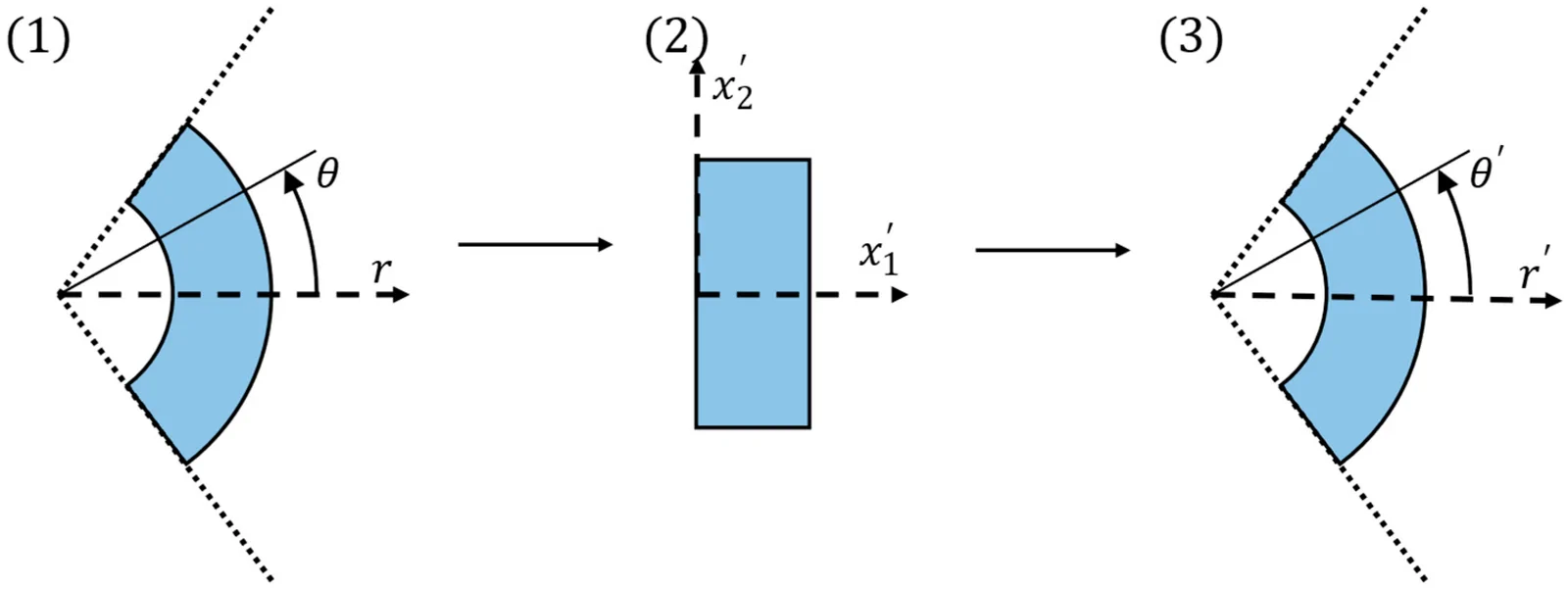
Two-step mapping from current radius to desired radius.

**Figure A2:**
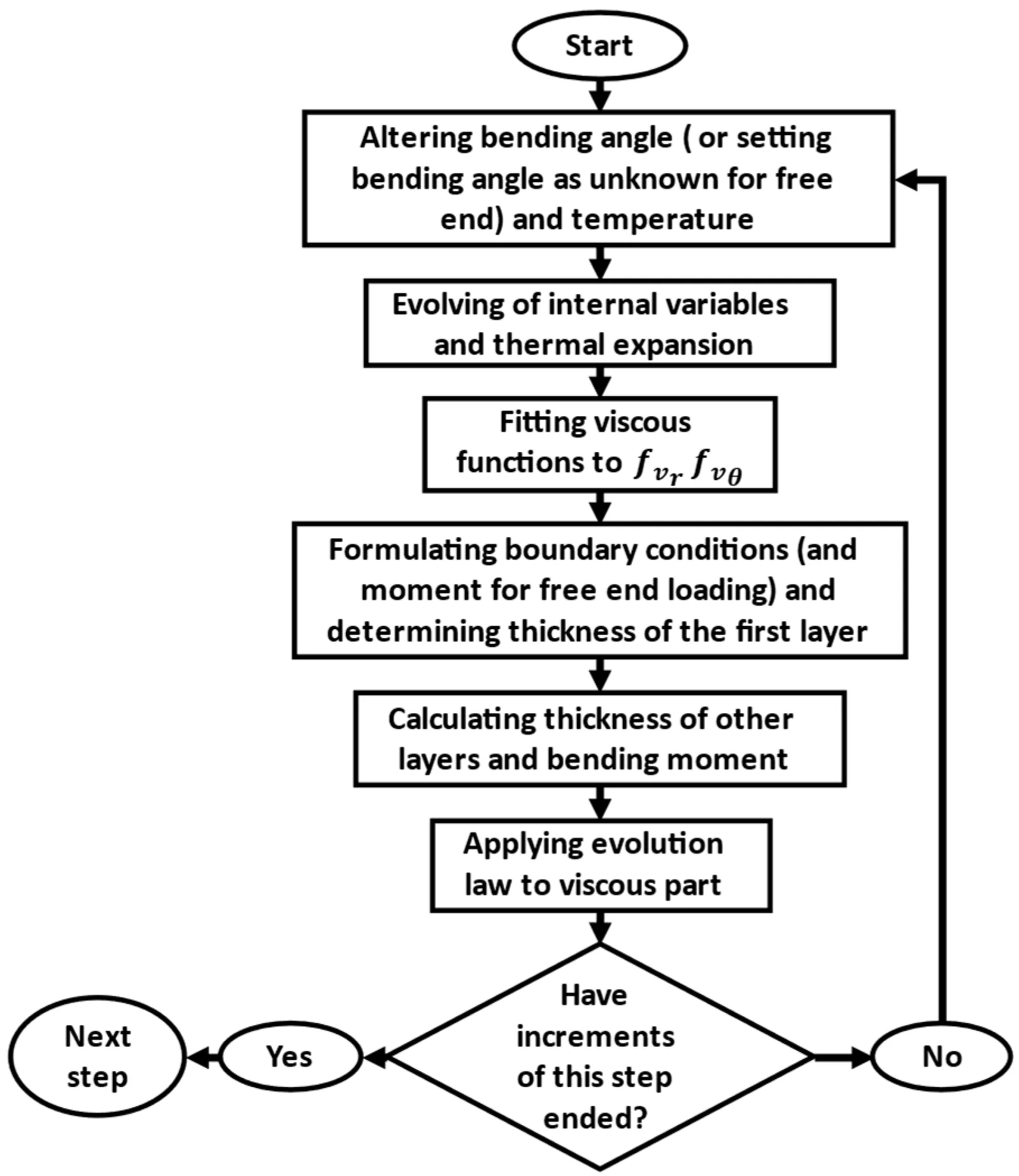
Flowchart of semi-analytical solution.

**Figure A3:**
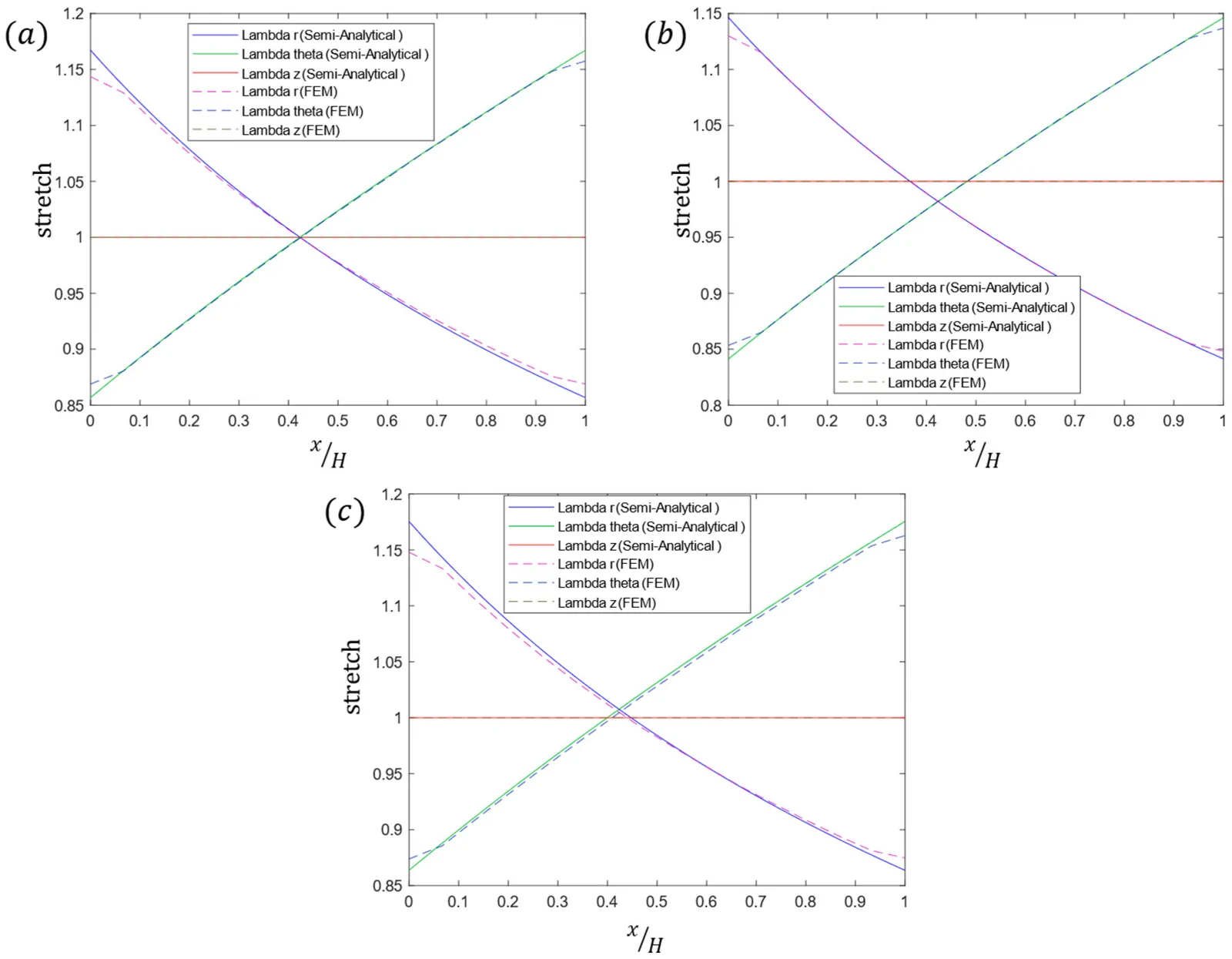
Principal stretches at (**a**) the end of the relaxation step, (**b**) the end of the cooling step, and (**c**) the end of the heating step.

**Figure A4:**
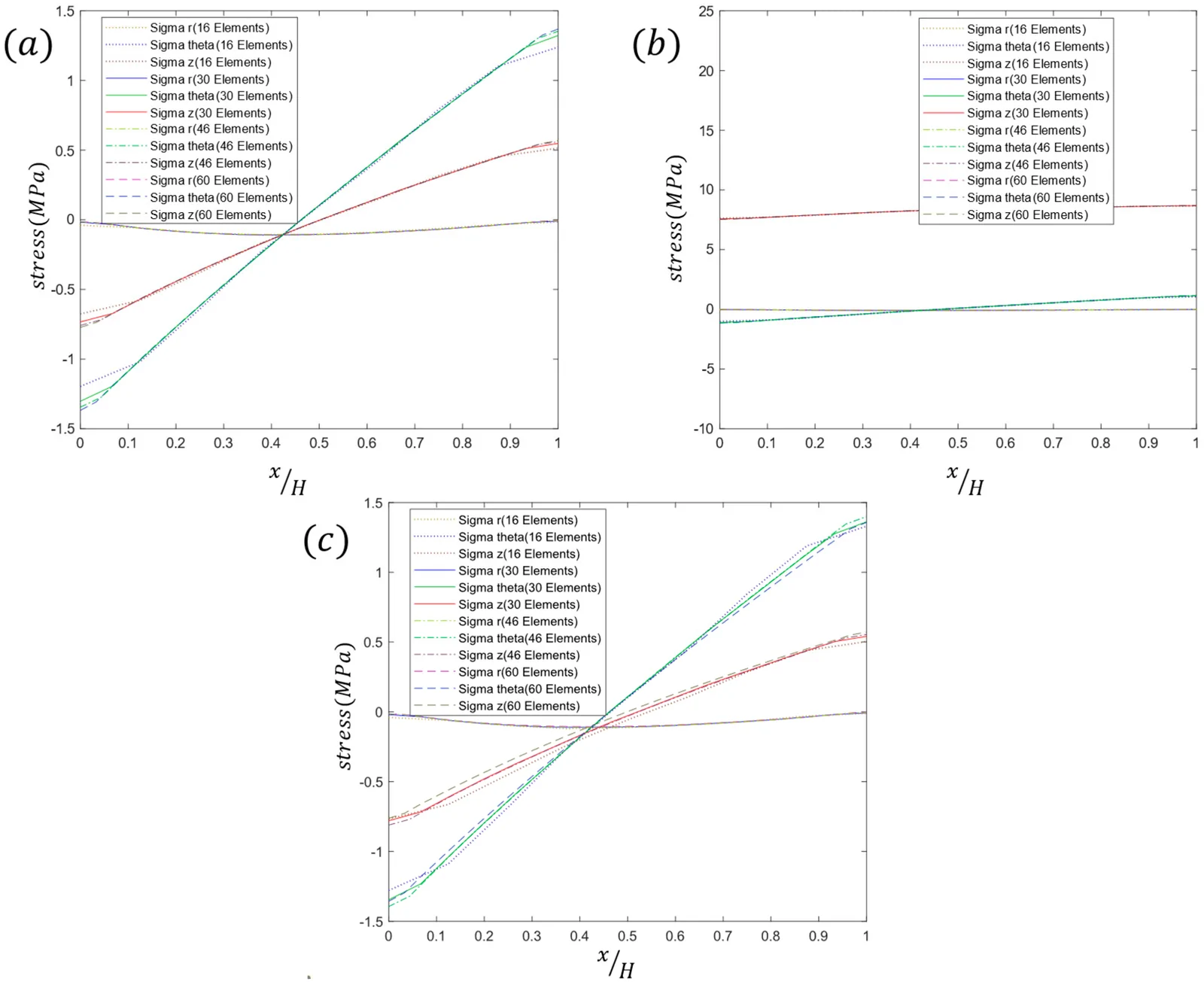
Principal stresses along the radial axis for Structure 5: (**a**) at the end of the post-loading relaxation step, (**b**) at the end of the cooling step, and (**c**) at the end of the force recovery cycle (H=10 mm).

## Data Availability

The original contributions presented in this study are included in the article. Further inquiries can be directed to the corresponding author(s).

## Appendix A

The evolution of the left Cauchy–Green tensor $B_{M}^{e}$ towards equilibrium is governed by the following nonlinear flow rule [58]:

(A1)
$$-\frac{1}{2}L_{v}B_{M}^{e}{B_{M}^{e}}^{-1}={\dot{\gamma }}^{v}n^{v}$$

The Lie derivative is given by $L_{v}B_{M}^{e}\;=\;-2\;F_{M}^{e}D_{M}^{v}F_{M}^{e^{T}}$. The flow direction is defined as $n^{v}=\frac{\sigma_{neq}}{\left\|\sigma_{neq}\right\|}$, and the flow rate is given by

(A2)
$${\dot{\gamma }}^{v}=\frac{s_{y}}{Q_{s}}T\tau_{s}sinh(\frac{Q_{s}\bar{\tau }}{Ts_{y}})$$

Here, $s_{y}$ is the activation stress, $\eta_{ref}$ and $Q_{s}$ are activation parameters, $\tau_{s}$ is the characteristic stress relaxation time, and $\bar{\tau }=\frac{\left\|\sigma_{neq}\right\|}{\sqrt{2}}=\sqrt{\frac{1}{2}\sigma_{neq}:\sigma_{neq}}$ is the equivalent stress. Considering the dependencies on temperature and structure, a new stress relaxation model is proposed here:

(A3)
$$\tau_{s}=exp(-\frac{1+\delta }{\log e}(1+\frac{T_{h}-T}{\eta_{ref}})-\left(1+sign\left(T-T_{r}\right)\right)\frac{{\left(T-T_{r}\right)}^{2}}{{\left(T-T_{r}\right)}^{2}+c})$$

(A4)
$$sign\left(x\right)=\left\{\begin{array}{c} 1\;x>0\\ 0\;x=0\\ -1\;x<0 \end{array}\right.$$

This model simulates plastic softening also by evolving the value of $s_{y}$, which is thoroughly discussed in Zeng’s work [43]. The parameter values of this constitutive model are specified in Table A1.

**Table A1:** Parameters of constitutive model [43].

Parameters	SMP-1	SMP-2	Physical Definitions
$\alpha_{r}(10^{-4}/K)$	6.9	6.3	Rubbery coefficient of volumetric thermal expansion
$\alpha_{g}(10^{-4}/K)$	2.34	2.4	Glassy coefficient of volumetric thermal expansion
$T_{g}(K)$	309	311	Glass transition temperature
$\tau_{T}^{0}$	1000	800	Characteristic structure relaxation time
θ(1/K)	0.7	0.7	Material temperature parameter
$T_{v}(K)$	303	303	Reference temperature
$\mu_{eq}(\mathrm{MPa})$	2	2	Shear modulus of equilibrium network
$\lambda_{L}$	4	4	Limiting chain stretch of equilibrium network
$E_{1},\;E_{2},\;E_{3}(\mathrm{MPa})$	447.16 1246.3 2.53	446.01 1203.4 5.03	Reference moduli of Prony series
$T_{1},\;T_{2},T_{3}(K)$	310.7 316.7 406.2	315.9 318.6 423.3	Reference temperatures of Prony series
$\alpha_{1},\;\alpha_{2},\alpha_{3}$	23.01 76.10 9.02	27.48 82.13 8.28	Reference coefficients of Prony series
$T_{h}(K)$	373	373	Reference high temperature for stress relaxation time
$\eta_{ref}$	15	15	Temperature dependence of the stress relaxation
$s_{0}(\mathrm{MPa})$	48	50	Initial value of activation stress
$s_{s}(\mathrm{MPa})$	28	30	Saturation value of activation stress
$Q_{s}(K)$	5300	5500	Activation parameter for viscous flow
$h_{0}(\mathrm{MPa})$	400	400	Parameter describing the yield variation
$T_{r}(K)$	315	318	Rubbery temperature
$c(K^{2})$	0.13	0.16	Glass transition temperature range

## Appendix B

As depicted in Figure A1, this mapping consists of two stages. First, the material points, defined by their radial coordinates, are mapped back to their initial positions (before deformation). At this stage, a point located at $r_{1}$ is mapped to zero. Subsequently, these initial points are then mapped forward to new points with updated radial coordinates.

As shown in Figure A1, to map points from State 1 to State 2, the following mapping is defined:

(A5)
$$x_{1}^{\prime }=\frac{1}{2\rho }\left(r^{2}-{\left(r_{1}\right)}^{2}\right)\;,\;x_{2}^{\prime }=\rho \theta$$

Subsequently, to transition points from State 2 to State 3, this mapping is applied:

(A6)
$$r^{\prime }=\sqrt{2\rho x_{1}^{\prime }+{\left(r_{1}^{\prime }\right)}^{2}}$$

Combining these two mappings yields the final composite mapping:

(A7)
$$r^{\prime }=\sqrt{r^{2}-{\left(r_{1}\right)}^{2}+{\left(r_{1}^{\prime }\right)}^{2}}$$

Here, $r^{\prime }$ represents the radius in the new coordinate system, and $r_{1}^{\prime }$ denotes the inner radius in the new coordinates. With the mapping established, the functions ${f_{v}}_{\theta }$ and ${f_{v}}_{r}$ can be formulated as follows:

(A8)
$${f_{v}}_{r}=\frac{c_{r}\rho }{\bar{\theta }\sqrt{r^{2}-{\left(r_{1}\right)}^{2}+{\left({r_{1}^{\prime }}_{r}\right)}^{2}}}$$

(A9)
$${f_{v}}_{\theta }=\frac{c_{\theta }\sqrt{r^{2}-{\left(r_{1}\right)}^{2}+{\left({r_{1}^{\prime }}_{\theta }\right)}^{2}}}{\rho }$$

In these expressions, $c_{r}$, ${r_{1}^{\prime }}_{r}$, $c_{\theta }$, and ${r_{1}^{\prime }}_{\theta }$ are coefficients of the defined functions. By adjusting these coefficients, the functions can be fitted to the viscous stretch at different radii. These fitted functions are then incorporated into the integral term of Equation (20). Integrating the derived functions with Equations (A8) and (A9), the non-equilibrium volumetric stress is obtained as follows:

(A10)
$$P_{neq}=\frac{\mu_{neq}}{J}\left(A-B-C\right)$$

(A11)
$$A=\frac{2{\left(J\right)}^{\frac{1}{3}}\log\left({\left({r_{i}^{\prime }}_{\theta }\right)}^{2}+r^{2}-{\left(r_{1}\right)}^{2}\right)}{c_{\theta }^{2}}$$

(A12)
$$B=\frac{{\left(J\right)}^{\frac{1}{3}}\left(2r^{2}\log\left(r\right)-{\left({r_{1}^{\prime }}_{r}\right)}^{2}+{\left(r_{1}\right)}^{2}\right)}{8c_{r}^{2}r^{2}}$$

(A13)
$$C={\left(\frac{{\left(J\right)}^{0.5}\sqrt{r^{2}-{\left(r_{1}\right)}^{2}+{\left({r_{1}^{\prime }}_{r}\right)}^{2}}}{2rc_{r}}\right)}^{2}$$

## Appendix C

The solution method flowchart is illustrated in Figure A2.

## Appendix D

At the start of each time increment, the deformation gradients *^t^**F*** and ^*t*+Δ*t*^***F***, the viscous deformation gradient ^*t*^$F_{M}^{v}$, the evolution coefficient for shear resistance ^*t*^$s_{y}$, and the internal variables $\delta_{i}$ (related to the thermal deformation gradient) are available.

The mechanical deformation gradient and the elastic deformation gradient are calculated as $F_{M}=FF_{T}^{-1}$ and F tMe=F tMF tMv−1, respectively.

The rates F˙ tMv and *^t^*${\dot{s}}_{y}$ are computed by substituting F tMe, F t, and sy t according to Section 2 and Appendix A. The estimated values for the viscous deformation gradient and the shear resistance evolution coefficient at t+Δt are calculated using the following relations:

F^ t+∆tMv=F tMv+F˙ tMv∆ts^y t+∆t=sy t+s˙y t∆t

The estimated elastic deformation gradient at t+Δt is computed as F tMe=F tF^ t+∆tMv−1. The rates F˙ t+∆tMv and s˙y t+∆t are subsequently calculated. The viscous deformation gradient and the shear resistance evolution coefficient at t+Δt are finally updated as

F t+∆tMv=F tMv+F˙ t+∆tMv+F˙ tMv2∆tsy t+∆t=sy t+s˙y t+∆t+s˙y t2∆t

It is important to note that at the beginning of the solution, the viscous deformation gradient F tMv is initialized as the identity matrix and is subsequently updated at each stage of the simulation. This approach ensures the initial state has no viscous deformation and allows the model to evolve accurately as the simulation progresses. The elastic deformation gradient at t+Δt is calculated as F t+∆tMe=F t+∆tMF t+∆tMv−1.

The Cauchy stress components ($\sigma_{eq}$, $\sigma_{neq}$, and P) at time t+Δt are computed by substituting F t+∆tMe into the relations in Equations (3)–(5). The total Cauchy stress σ t+∆t at time t+Δt is obtained by summing the components σ t+∆teq, σ t+∆tneq, and P t+∆t according to $\sigma =\sigma_{eq}+\sigma_{neq}+P$. Finally, F t+∆tMv, sy t+∆t, and the internal variables $\delta_{i}$ are stored, and σ t+∆t is passed back to Abaqus.

## Appendix E

Figure A3 displays the principal stretches at three key instants: the end of the relaxation step, the end of the cooling step, and the final moment of the heating step, comparing the results of the semi-analytical method and the explicit FEM. As is evident from the figure, a strong correlation exists between the results, confirming the correct definition of stretches in the semi-analytical approach and thereby validating the accuracy of the proposed methodology.

Figure A4 depicts the distribution of principal stresses along the radial axis, obtained using the explicit FEM, for three distinct time intervals: the end of the post-loading relaxation phase, the conclusion of the cooling stage, and the final step of the bending force recovery cycle. These plots were generated using different mesh densities to examine the influence of element size on the results.
